# Influence of Referral Pathway on Ebola Virus Disease Case-Fatality Rate and Effect of Survival Selection Bias

**DOI:** 10.3201/eid2304.160485

**Published:** 2017-04

**Authors:** Frauke Rudolf, Mads Damkjær, Suzanne Lunding, Kenn Dornonville de la Cour, Alyssa Young, Tim Brooks, Tom Sesay, Alex P. Salam, Sharmistha Mishra, Merete Storgaard

**Affiliations:** Aarhus University Hospital, Skejby, Denmark (F. Rudolf, M. Storgaard); GOAL Global, Port Loko, Sierra Leone (M. Damkjær, S. Lunding, K.D. de la Cour, A. Young, A.P. Salam, M. Storgaard);; Danish Armed Forces Health Services, Brabrand, Denmark (F. Rudolf, M. Damkjær, S. Lunding, K.D. de la Cour, M. Storgaard);; Hans Christian Andersen Children’s Hospital, Odense, Denmark (M. Damkjær);; Copenhagen University Hospital, Hillerod, Denmark (S. Lunding);; Odense University Hospital, Odense (K.D. de la Cour);; GOAL Global, Freetown, Sierra Leone (A. Young);; Public Health England, Salisbury, UK (T. Brooks);; Sierra Leone Ministry of Health and Sanitation, Freetown (T. Sesay);; Wohl King’s College Clinical Neuroscience Institute, London, UK (A.P. Salam);; University of Toronto St. Michael’s Hospital, Toronto, Ontario, Canada (S. Mishra);

**Keywords:** Ebola virus disease, Ebola virus, Ebola treatment centers, Ebola isolation centers, case-fatality rate, referral pathway, selection bias, Sierra Leone, viruses, EVD, zoonoses

## Abstract

Case-fatality rates in Ebola treatment centers (ETCs) varied widely during the Ebola virus disease (EVD) outbreak in West Africa. We assessed the influence of referral pathway on ETC case-fatality rates with a retrospective cohort of 126 patients treated at the Mathaska ETC in Port Loko, Sierra Leone. The patients consisted of persons who had confirmed EVD when transferred to the ETC or who had been diagnosed onsite. The case-fatality rate for transferred patients was 46% versus 67% for patients diagnosed onsite (p = 0.02). The difference was mediated by Ebola viral load at diagnosis, suggesting a survival selection bias. Comparisons of case-fatality rates across ETCs and clinical management strategies should account for potential survival selection bias.

As of February 14, 2016, the 2014–2016 outbreak of Ebola virus in West Africa had resulted in >14,000 cases of Ebola virus disease (EVD) and ≈4,000 deaths in Sierra Leone (*1*). The country’s strategy for managing the outbreak and isolating patients included decentralized Ebola treatment centers (ETCs) and Ebola isolation centers (EICs), which were also known as community care centers and holding centers (*2*,*3*). EICs were transitional facilities meant for admission and isolation of patients who were awaiting results of Ebola diagnostic testing (real-time PCR) and provision of basic care (e.g., administration of oral rehydration solution) (*2*). EIC patients with Ebola virus–negative test results were discharged, and those with positive results were transferred to an ETC. In contrast to EICs, ETCs could care for patients suspected of having and those confirmed to have EVD without transfer of patients between facilities. EICs were initially designed to address a shortfall in ETC bed capacity, although their use continued even as ETC bed capacity increased during the outbreak (*1*).

Recent studies on EVD clinical outcomes (*2*,*4*–*7*) demonstrate considerable variability in case-fatality rates (37%–74%) and call for further analyses to understand the reason(s) for this variability. Predictors of higher case-fatality rate after ETC admission are age (*4*–*6*,*8*) and higher viremia at diagnosis (*9*,*10*) and, less consistently, longer symptom duration before admission (*4*–*6*,*8*,*9*); clinical presentation with confusion, diarrhea, and conjunctivitis (*4*–*6*,*8*); and biochemical evidence of kidney injury, hepatitis, or both (*5*). One study reported early EVD-associated deaths (i.e., in the community) with a case-fatality rate of 24% before ETC transfer (*9*). None of these studies examined the care pathway of EVD patients or the extent to which direct admission to an ETC versus transfer from an EIC influenced case-fatality rates measured in ETCs.

We sought to investigate whether referral pathway had any influence on case-fatality rate. We specifically sought to determine whether there was a statistically significant difference in case-fatality rate between EVD patients admitted directly to the ETC compared with patients first admitted to an EIC and subsequently transferred to the ETC after confirmation of EVD status.

## Methods

### Study Setting

We conducted a retrospective cohort study on all patients with EVD admitted to the Mathaska ETC in Port Loko district, Sierra Leone, during December 12, 2014–March 14, 2015 (i.e., from the time the ETC opened until the first author of this article left Sierra Leone). GOAL Global (https://www.goalglobal.org/) ran the ETC with national and international staff, and the ETC received patients from Port Loko and the neighboring district, Kambia. The ETC received patients via 2 referral paths: 1) patients transferred from an EICs after testing positive for Ebola virus (cohort 1, confirmed cases); and 2) patients admitted after meeting the Sierra Leone Ministry of Health and Sanitation (MoHS) case definition of presumed EVD detected through active monitoring of contacts in quarantine or through passive surveillance in communities and non-EVD healthcare facilities (cohort 2, suspected or probable cases). We included cohort 2 patients in the study only if they were confirmed to have EVD (i.e., Ebola-positive real-time PCR results) after admission to the ETC.

All cases of EVD during the study period in Port Loko and Kambia were confirmed by PCR testing of blood; testing was performed at the Public Health England reference laboratory in Port Loko. Patients received symptomatic treatment according to World Health Organization and MoHS guidelines (*11*). The Sierra Leone Ethics and Scientific Review Committee approved the study.

### Data and Analyses

We used EpiData version 3.1 software (EpiData Association, Odense, Denmark) to extract and compare the following data for directly admitted and transferred patients: demographic data (age, sex); clinical data (time from symptom onset to EVD test, death vs. survival in ETC); laboratory data (PCR results, cycle threshold [C_t_]); and referral and admission data from clinical charts. For all patients, we used the C_t_ from the initial positive blood sample (i.e., for cohort 1, the blood sample was from the EIC). The C_t_ is inversely proportional to the level of virus in the blood sample (*12*). We verified clinical documentation of referral source and cohort 1 classification from MoHS surveillance data and by checking the EVD test date against the ETC admission date. While waiting for PCR results, 6 EIC patients were admitted to the ETC’s ward for patients with suspected or probable EVD. To be consistent in our analysis, we considered those patients directly admitted patients (cohort 1).

We calculated the time from symptom onset to EVD test by subtracting the self-reported symptoms-onset date from the first EVD test (which occurred on the date of admission to an EIC or ETC). We assessed the number of symptoms at admission as well as the stage of disease (i.e., the presence of mild influenza-like symptoms, wet symptoms [i.e., diarrhea or vomiting, or both], or hemorrhagic symptoms). Case-fatality was recorded as a death in the ETC. Survival among patients with confirmed EVD was defined as a resolution of viremia, as confirmed by an Ebola virus–negative PCR result. We assessed for differences in proportions by using the χ^2^ test for categorical variables with >5 observations/cell in a frequency table (death by cohort, admission stage by cohort, case-fatality rate by cohort). We used the *t*-test to compare continuous variables if normally distributed (age distribution between cohorts); we applied the Wilcoxon–Mann–Whitney rank test if nonnormal distribution was found (interval from symptom onset to EVD testing between cohorts, interval from symptom onset to ETC admission between cohorts, C_t_ between cohorts, association between low C_t_ and increased case-fatality rate). To graphically represent the time to death, we used Kaplan-Meier survival curves. To assess difference in fatality rates between transferred and directly admitted patients, we used Cox proportional hazards analysis adjusted for C_t_ value; no significant deviations from the proportional hazards assumption were found. We used Stata 12 (StataCorp LLC, College Station, TX, USA) to analyze data.

## Results

During the study period, 227 patients were transferred and admitted to the ETC; 128 of these patients had EVD. Of those 128 patients, 126 were included in the study. The 2 excluded EVD patients were transferred to another ETC, and their outcomes were unknown. Female patients comprised 53% of the patients. The median age of patients was 30 years (interquartile range [IQR] 18–42); 27 patients were <15 years of age.

The overall EVD case-fatality rate at Mathaska ETC was 59% (74/126 patients). The case-fatality rate was highest among children <2 years of age (67%) and persons >35 years of age (78%). Of the 74 patients who died, 72 (97%) died within 9 days of ETC admission (Figure).

**Figure:**
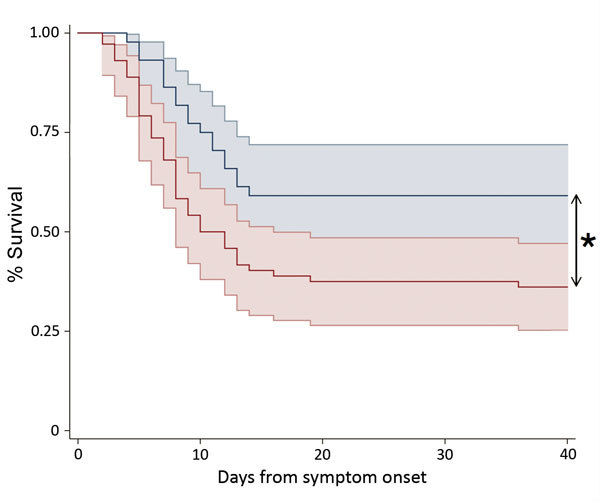
Kaplan-Meier survival plot stratified by referral pathway for patients admitted directly to an Ebola treatment center (ETC) with confirmed Ebola virus disease (cohort 1, blue line) and for patients diagnosed at the ETC (cohort 2, red line). Plots show the percentage of patients surviving as a function of time (days) from reported symptom onset. Shaded areas indicate 95% CIs. *p<0.05.

Cohort 1 comprised 48 patients who were transferred to Mathaska ETC from EICs (n = 45) or other local ETCs (n = 3) with a confirmed EVD diagnosis. Cohort 2 comprised 78 patients: 16 from quarantine, 1 from a non-EVD hospital, 6 from EICs in Kambia district, 1 from another ETC, and 53 who were referred through community surveillance. The referral pathway was missing for 1 patient. The age distribution was similar between cohorts (p = 0.2) (Table). The time from symptom onset to admission at the ETC was shorter for cohort 2 patients than cohort 1 patients (4 vs. 6 days; p<0.001), and the C_t_ at diagnosis was higher among cohort 1 patients than cohort 2 patients (23 vs. 20; p<0.001).

**Table:** Demographic and epidemiologic differences between 2 patient cohorts in a study of the sources of variability in case-fatality rates in Ebola treatment centers, Sierra Leone, 2014–2016*

Variable	Cohort 1, n = 48	Cohort 2, n = 78	p value
Case-fatality rate, %	46	67	0.02
Age, median, IQR	29 (14–40)	34 (20–45)	0.2
Children <15 years of age, no. (%)	12 (44)	15 (56)	0.4
Days from symptom onset to EVD testing (IQR)	4 (2–5)	4 (3–6)	0.7
Days from symptom onset to admission at ETC, median (IQR)	6 (4–7)	4 (2–5)	<0.001
C_t_, median (IQR)†	23 (21–26)	20 (18–23)	<0.001

*Cohort 1 consisted of patients admitted directly to the ETC (Ebola treatment center) with confirmed EVD (Ebola virus disease); cohort 2 consisted of patients admitted directly to the ETC, where they were subsequently diagnosed with EVD. C_t_, cycle threshold; IQR, interquartile range. †Obtained from first blood sample drawn.

The median duration of symptoms before EVD testing was similar between cohorts 1 and 2 (median 4 [IQR 2–5] days vs. 4 [IQR 3–6] days; p = 0.7) (Table). A lower C_t_ was associated with an increased case-fatality rate (p<0.001). Neither the quantity of symptoms (mean 6.5 [cohort 1] vs. 6.2 [cohort 2]; p = 0.3) nor the distribution of patients according to severity stages differed significantly between cohorts 1 and 2 (p = 0.8).

The case-fatality rate was lower for cohort 1 (EVD confirmed before transfer) than cohort 2 (46% vs. 67%; p = 0.02) (Table). After we adjusted for C_t_ at diagnosis, the case-fatality rate was no longer significantly different between cohorts 1 and 2 (p = 0.2).

## Discussion

Understanding sources of variability in observed case-fatality rates during the 2014–2016 Ebola virus outbreak in West Africa is essential for interpreting case-fatality rate as part of routine monitoring of a clinical program (*1*) and for evaluating the effect of clinical interventions. We investigated whether patients transferred to Mathaska ETC had a lower case-fatality rate than patients directly admitted to the ETC, and our results show that referral pathway does influence the case-fatality rate. This finding confirms the observations from previous studies showing that virus load and patient age are associated with EVD case fatality (*4*).

In Sierra Leone, 5 ETCs with different referral pathways reported different case-fatality rates. Among EVD patients admitted and diagnosed onsite at Kenema Government Hospital early in the outbreak, the case-fatality rate was 74% (64 deaths/87 patients) (*6*). The Médecins Sans Frontières ETC in Kailahun admitted and diagnosed patients on-site and reported a case-fatality rate of 51% (270 deaths/525 patients) (*4*). In Bo, the case-fatality rate was 66% (142/216) among all confirmed EVD patients detected in the community during the study period, but it was 40% (49/123) among the detected EVD patients who survived until admittance to an ETC (*9*). In contrast, the Save the Children ETC in Kerry Town, Sierra Leone, had a case-fatality rate of 37% (55 deaths/150 patients) and was equipped to provide a higher level of care (additional diagnostics) but received only confirmed patients from EICs (*5*). Among the 85 EVD patients admitted to the EIC in the Jui Government Hospital in Sierra Leone, the case-fatality rate was 60%, although it was unclear whether the deaths occurred in the EIC or in the ETC to which confirmed patients were transferred (*7*).

We found that the influence of referral pathways on the estimated case-fatality rate at Mathaska ETC was probably mediated by differences in virus load at diagnosis. This finding supports the hypothesis that differences in observed case-fatality rates by referral pathway are probably due to survival selection bias rather than differences in patient care at individual ETCs. We did not measure case-fatality rates in the EICs. Thus, we cannot infer the role of EIC versus ETC on case-fatality rate before EVD confirmation and transfer to EVD-confirmed wards. Furthermore, although the difference in virus load among the 2 cohorts suggests that the transferred patients were in recovery, there was no difference in the number of symptoms nor in the severity of disease when patients were admitted to the ETC. We did not, however, assess the degree of the individual symptoms, and that information might have added clarity.

Our data, along with the case-fatality rates reported for other ETCs in Sierra Leone (*4*,*5*), suggest that if the referral pathway (i.e., time spent in EICs) is long, patients may die before getting tested for EVD disease. Thus, EVD patients transferred to the ETC represent a different patient population than those diagnosed on-site. Our findings of possible survivor selection bias are consistent with findings in previous reports showing a higher case-fatality rate among patients who were admitted early after symptom onset (*4*,*5*), an initially counter-intuitive finding, given the provision early supportive management. In Kailahun, patients who traveled long distances to reach the ETC had a lower case-fatality rate than those who traveled shorter distances (*4*). Although, as pointed out by Hunt et al. (*5*), reported symptom onset date is subject to recall bias. Thus studies of clinical predictors and comparisons of case-fatality rates across ETCs must account for potential survivor selection bias. Symptom-onset date is prone to recall and social desirability bias, but referral pathways are an objective indicator of potential differences in patient populations admitted to ETCs. From a clinical perspective in the ETC, measuring and making decisions based on anticipated efficacy of supportive management or experimental drugs must account for these differences in patients. A key social mobilization message during the West Africa outbreak was the importance of early diagnosis and treatment to save lives (not just prevent transmission), drawing on experience and evidence from other infectious diseases with similar end-organ effects. Invasive monitoring and careful fluid management probably contributed to the low case-fatality rates observed in the study in Kerry Town, but as the authors noted, the study population was subject to selection bias (*5*), which limits the generalizability of care-associated predictors of outcome. Rigorous study of all patients with confirmed EVD and estimates of case-fatality rate at each point in the referral pathway (community, EIC, ETC) are needed to disentangle survival selection bias from the effect of early care and care-associated predictors of case-fatality rate.

In conclusion, case-fatality rates across ETCs may depend on which patients are referred to the facilities and, thus, the distribution of known predictors, such as age and virus load. Referral pathways and the potential for survival selection bias should be accounted for when comparing case-fatality rates between studies, ETCs, and interventions and when planning and evaluating future clinical trials.
